# Effects of COVID-19 pandemic on colorectal cancer surgery

**DOI:** 10.1590/1516-3180.2021.0357.R1.30062021

**Published:** 2021-09-27

**Authors:** Mikail Uyan, Ali Özdemir, Süleyman Kalcan, Kadir Tomas, Gökhan Demiral, Ahmet Pergel, İsmail Alper Tarım

**Affiliations:** I MD, PhD. Assistant Professor, Department of General Surgery, Recep Tayyip Erdoğan University Medical Faculty, Rize, Turkey.; II MD, PhD. General Surgeon, Department of General Surgery, Recep Tayyip Erdoğan University Medical Faculty, Rize, Turkey.; III MD, PhD. Assistant Professor, Department of General Surgery, Recep Tayyip Erdoğan University Medical Faculty, Rize, Turkey.; IV MD, PhD. General Surgeon, Department of General Surgery, Recep Tayyip Erdoğan University Medical Faculty, Rize, Turkey.; V MD, PhD. Associate Professor, Department of General Surgery, Recep Tayyip Erdoğan University Medical Faculty, Rize, Turkey.; VI MD, PhD. Professor, Department of General Surgery, Recep Tayyip Erdoğan University Medical Faculty, Rize, Turkey.; VII MD, PhD. Assistant Professor, Department of General Surgery, Ondokuz Mayıs University Medical Faculty, Samsun, Turkey.

**Keywords:** COVID-19, Colorectal neoplasms, Surgery [subheading], COVID-19 pandemic, Emergency surgery, Colorectal cancer

## Abstract

**BACKGROUND:**

The coronavirus disease-19 (COVID-19) pandemic has changed the course of diseases that require emergency surgery.

**OBJECTIVE:**

To evaluate the effect of the COVID-19 pandemic on colorectal cancer disease stage.

**DESIGN AND SETTING:**

Retrospective analysis in the city of Rize, Turkey.

**METHODS:**

This was a comparative analysis on two groups of patients with various symptoms who underwent surgical colorectal cancer treatment. Group 1 comprised patients operated between March 11, 2019, and December 31, 2019; while group 2 comprised patients at the same time of the year during the COVID-19 pandemic.

**RESULTS:**

Groups 1 and 2 included 56 and 48 patients, respectively. The rate of presentation to the emergency service was higher in Group 2 (P < 0.02). The stage of the pathological lymph nodes and the rate of liver metastasis was higher in Group 2 (P < 0.004 and P < 0.041, respectively). The disease stage was found to be more advanced in Group 2 (P < 0.005). The rate of postoperative complications was higher in Group 2 (P < 0.014).

**CONCLUSION:**

The presentation of patients with suspicious findings to the hospital was delayed, due both to the fear of catching COVID-19 and to the pandemic precautions that were proposed and implemented by healthcare authorities worldwide. Among the patients who presented to the hospital with emergency complaints and in whom colorectal cancer was detected, their disease was at a more advanced stage and thus a higher number of emergency oncological surgical procedures were performed on those patients.

## INTRODUCTION

Cancer is a problem with worldwide importance and is the second leading cause of death globally.^1^ Colorectal cancers, with an incidence of 9%, are the fourth among all cancers, following lung, breast and prostate cancer. This rate varies between different countries and ethnicities.^2^ The countries with the highest incidence rates include Australia, New Zealand, Canada, the United States and parts of Europe. The countries with the lowest risk include China, India and parts of Africa and South America.^3^

During the last 30 years, the incidence of colorectal cancer has decreased from 66.3 to 45.3 per 100,000 population in the United States.^4^ The most important cause of this reduction has been the development and extensive implementation of colorectal cancer screening programs, which have made a positive contribution to the prognosis for the disease, through early detection and diagnosis.^5^

The first cases of pneumonia caused by severe acute respiratory syndrome coronavirus 2 (SARS-CoV-2) that were reported were in Wuhan, China, in December 2019.^6^ A global pandemic was declared by the World Health Organization on March 11, 2020.^7^ Coronavirus disease-19 (COVID-19) has seriously jeopardized the health of the whole world, but especially that of healthcare workers.^8^

The number of cases of all other diseases presenting to hospitals and emergency services decreased significantly after cases of COVID-19 began to be seen on March 11. A decision was made by the Turkish Ministry of Health on March 17, to delay all elective surgical procedures while pandemic precautions were continuing to be implemented. In addition to these precautions, the fear of contracting COVID-19 also impacted the rate of access to the country’s healthcare services.^9^

This raises various questions, such as: “If patients’ presentations to hospitals were delayed despite the presence of suspicious symptoms, were the procedures of colorectal cancer surgery more complicated during the COVID-19 pandemic; did ­COVID-19 affect the staging of colorectal cancer; and were the rates of emergency colorectal surgery, postoperative complications and mortality changed by the COVID-19 pandemic?”

## OBJECTIVE

The aim of this study was to evaluate the effects of the pandemic precautions suggested and applied by healthcare authorities worldwide and the impact of the fear of becoming infected by SARS-CoV-2, on delay in detection and diagnosis of colorectal cancer, disease stage at the time of diagnosis and emergency surgery rates, during the COVID-19 pandemic.

## METHODS

This study was conducted retrospectively, after obtaining ethics board approval from the Ministry of Health (2021-01-06T14_53_45) and from the Ethics Board of the School of Medicine of Recep Tayyip Erdoğan University (approval number: 2021/27; date: February 4, 2021).

This study involved comparative analysis between patients who were treated for colorectal cancer during the COVID-19 pandemic and patients treated for colorectal cancer one year before the pandemic but at the same time of year. The patients were divided into two groups. Group 1 included patients who were treated for colorectal cancer between March 11, 2019, and December 31, 2019, while Group 2 included patients treated for colorectal cancer between March 11, 2020, and December 31, 2020, during the COVID-19 outbreak.

The patients’ age and gender, emergency status of the operations, treatment modality applied and surgical procedure, localization of the tumor, histopathological result from the surgical specimen, disease stage, postoperative complications and duration of postoperative stay were recorded separately in Group 1 and Group 2. The differences between the groups were compared.

An analysis on the data was made using PASW Statistics (version 18.0; SPSS Inc., Chicago, Illinois, United States). The Mann-Whitney U test was used to analyze continuous variables. Variables such as age and gender were compared with Student’s t test. Pearson’s chi-square test was used to evaluate numerical data between groups. The level of statistical significance was set at a P-value of less than 0.05.

## RESULTS

A total of 104 patients were included in this study. Among these patients, 63 (60.5%) were male and 41 (39.5%) were female, with a mean age of 64 years. Groups 1 and 2 included 56 and 48 patients, respectively. There were 32 males (57%) and 24 females (43%) in Group 1; while Group 2 comprised 31 males (64%) and 17 females (36%). No statistical significance in terms of gender was found between the groups (P = 0.439). No significant difference in mean age was found between the groups, either: Group 1, 64.9 years (range = 41-89); Group 2, 63.2 years (range = 22-90; P = 0.492 (Table 1).

**Table 1.** Demographic and perioperative data of the patients

	Group 1	Group 2	P
	(2019)	(2020)	
Number of patients, n (%)	56 (54)	48 (46)	
Gender, n (%)			
Male	32 (57)	31 (64)	0.439
Female	24 (43)	17 (36)	
Age, mean (minimum-maximum)	64.9 (41-89)	63.2 (22-90)	0.492
Emergency/elective, n (%)			
Emergency	13 (23)	25 (52)	0.02
Elective	43 (77)	23 (48)	
Reason for surgery, n (%)			
Elective		23 (48)	
Ileus	43 (77)	16 (33)	
Tumor perforation	10 (18)	8 (17)	
Gastrointestinal bleeding	3 (5)	1 (2)	
Treatment modality, n (%)			
Laparotomy	40 (71)	39 (81)	0.243
Laparoscopy	16 (29)	9 (19)	
Surgical procedure, n (%)			
Hemicolectomy		24 (50)	0.663
Low anterior resection	30 (54)	20 (42)	
Abdominoperineal resection	21 (37)	3 (6)	
Total colectomy	5 (9)	1 (2)	
Tumor localization, n (%)			
Cecum	5 (8)	11 (23)	0.511
Ascending colon	15 (27)	1 (2)	
Transverse colon	1 (2)	2 (4)	
Descending colon	8 (14)	1 (2)	
Sigmoid colon	2 (4)	9 (19)	
Rectosigmoid	3 (5)	13 (27)	
Rectum	22 (40)	11 (23)	
Length of stay, mean (minimum-maximum)	9.3 (1-54)	10.8 (1-56)	0.332
Postoperative complications, n (%)			
Yes	11 (20)	20 (42)	0.014
No	45 (80)	28 (58)	
Complications, n (%)			
Wound site infection			
Ileus		7 (14.6)	
Hematoma/bleeding	5 (8.9)	2 (4.2)	
Anastomotic leak	1 (1.8)	1 (2.1)	
Evisceration	2 (3.6)	1 (2.1)	
Extraperitoneal complications (lung and cardiac problems)	3 (5.4)	9 (18.8)	
Mortality, n (%)			
Yes	3 (5)	4 (8)	0.209
No	53 (95)	44 (92)	

The numbers of emergency and elective operations were 13 (23%) and 43 (77%) in Group 1, respectively; and 25 (52%) and 23 (48%) in Group 2, respectively. The number of patients undergoing emergency surgery was significantly higher in Group 2 (P = 0.02) (Table 1). Among the patients undergoing emergency surgery, the indication for the operation was ileus in 10 (18%) and tumor perforation in three (5%) in Group 1; while in Group 2 it was ileus in 16 (33%), tumor perforation in eight (17%) and gastrointestinal (GIS) bleeding in one (2%) (Table 1).

Laparotomy and laparoscopic surgery was performed in 40 (71%) and 16 patients (29%), respectively in Group 1; while 39 patients (81%) underwent laparotomy and nine patients (19%) underwent laparoscopic procedures in Group 2 (P = 0.243) (Table 2). No significant difference in the surgical procedure performed and the localization of the tumor was found (P = 0.663 and P = 0.511, respectively) (Table 1).

**Table 2.** Pathological characteristics and postoperative data

	Group 1	Group 2	P
	(2019)	(2020)	
	n (%): 56 (54)	n (%): 48 (46)	
Pathological diagnosis, n (%)			
Adenocarcinoma	56 (100)	46 (96)	0.304
Medullary carcinoma		1 (2)	
Neuroendocrine tumor		1 (2)	
Pathological tumor stage, n (%)			
T1	3 (5)	0 (0)	0.240
T2	6 (10)	8 (17)	
T3	34 (62)	25 (52)	
T4	13 (23)	15 (31)	
Pathological lymph node stage, n (%)			
N0	39 (70)	21 (44)	0.004
N1	4 (7)	15 (31)	
N2	13 (23)	12 (25)	
Pathological stage, n (%)			
Stage 1	9 (16)	5 (10)	0.005
Stage 2	29 (52)	11 (23)	
Stage 3	13 (23)	21 (44)	
Stage 4	5 (9)	11 (23)	
Liver metastasis, n (%)			
Yes	4 (7)	10 (21)	0.041
No	52 (93)	38 (79)	
Synchronous tumor, n (%)			
Yes	4 (7)	2 (4)	0.516
No	52 (93)	46 (96)	
Peritoneal carcinomatosis, n (%)			
Yes	2 (4)	1 (2)	0.651
No	54 (96)	47 (98)	
Colostomy, n (%)	21(37)	22 (46)	0.390

The mean duration of the postoperative hospital stay was 9.3 days and 10.8 days in Group 1 and Group 2, respectively, with no difference between the groups (P = 0.332) (Table 1).

Postoperative complications occurred in 11 (20%) and 20 patients (42%) in Groups 1 and 2, respectively, and the rate of postoperative complications was significantly higher in Group 2 (P = 0.014) (Table 1). The complications seen were hematoma in one patient (1.8%), ileus in five (8.9%), anastomotic leakage in two (3.6%) and extraperitoneal complications in three (5.4%) in Group 1; while they were wound site infection in seven (14.6%), ileus in two (4.2%), evisceration in one (2.1%), anastomotic leakage in one (2.1%) and extraperitoneal complications in nine (18.8%) in Group 2 (Table 1).

Mortality in the early period was seen in three cases (5%) and four cases (8%) in Group 1 and Group 2, respectively with no difference between the groups (P = 0.209) (Table 1).

The histopathological diagnosis was adenocarcinoma in all patients in Group 1 (100%); while in Group 2 it was adenocarcinoma in 46 patients (96%), medullary carcinoma in one patient (2%) and neuroendocrine carcinoma in one patient (2%). No difference in terms of histopathological diagnosis was found between the groups (P = 0.304) (Table 2).

No significant difference in the pathological tumor stage (T) was found between the two groups (P = 0.240) (Table 2). However, the pathological lymph node stage (N) was significantly higher in Group 2 (N0 70% versus 44%, respectively; N1 7% versus 31%, respectively; and N2 23% versus 25%, respectively; P = 0.004) (Table 2).

The disease stage at presentation was stage 1 in nine patients (16%), stage 2 in 29 (52%), stage 3 in 13 (23%) and stage 4 in five (9%) in Group 1; while it was stage 1 in five patients (10%), stage 2 in 11 (23%), stage 3 in 21 (44%) and stage 4 in 11 (23%) in Group 2. Patients in Group 2 were diagnosed at a later stage than were patients in Group 1 (P = 0.005) (Table 2). Similarly, the rate of liver metastasis was also significantly different between the groups, in favor of Group 2. Liver metastasis was present in four patients (7%) and 10 patients (21%) in Group 1 and Group 2, respectively (P = 0.041) (Table 2). The presence of synchronous tumors and peritoneal carcinomatosis was similar in the two groups (P = 0.516 and P = 0.651, respectively) (Table 2).

## DISCUSSION

The COVID-19 outbreak seriously changed the structure of healthcare systems globally. Many surgical associations recommended that elective surgical operations should cease and that permission should only be granted for presentation of patients with emergency conditions, while cancer surgery could continue.^10^^,^^11^^,^^12^

Many papers have highlighted the precautions that would need to be taken while carrying out emergency and elective surgical procedures during the COVID-19 pandemic.^13^^,^^14^^,^^15^^,^^16^ Consequent to the COVID-19 pandemic, there have been many new changes regarding re-planning of departments within hospitals, with measures applied to reduce the risk of transmission to the hospital staff, and emphasis on the importance of using of personal protective equipment.^17^^,^^18^ These changes have greatly extended operating times and the time taken to clean operating rooms between surgeries. Moreover, given that surgeons and operating room workers do not want to endanger their own health, they may have even been avoiding operations on cancer cases unless emergency conditions occur.

At the same time, the protective equipment that surgeons and the entire operating team had to use due to the pandemic restricted the movement of the entire operating team, especially surgeons. Benitez et al. reported that the use of personal protective equipment adversely affected the performance of the surgeons.^19^

Here, in the present study, our aim was to evaluate the situation regarding delayed colorectal cancer disease diagnosis and its consequences. In our study, the age and gender of the patients operated on were similar in the two groups (Table 2).

Syllaios et al. reported that the number of emergency colorectal cancer surgeries increased by approximately 30% and that the number of minimally invasive surgical procedures decreased during the COVID-19 outbreak.^20^ The rate of occurrence of emergency surgery was found to be significantly higher in Group 2 in the present study (Table 2). Hence, it can be stated that patients with suspicious findings probably ignored their situation and preferred to wait at home. This result raises two possibilities: either the patients feared contracting the disease through exposure to the outside environment and consequently stayed at home, or delays in accessing healthcare services and in getting a diagnosis occurred due to pandemic precautions.

Algorithms relating to the approach to be taken in cases of emergency surgery have changed many times during the pandemic. For example, conservative approaches such as antibiotic therapy instead of surgery in acute appendicitis cases and application of laparotomy instead of laparoscopy have been preferred.^21^^,^^22^^,^^23^ Laparotomy was performed at a rate of 81% during the COVID-19 period with no significant difference between the groups, according to the results from our study (P = 0.243) (Table 1).

Comorbidities and disease stage have been investigated in order to determine the outcomes from colorectal surgical treatment.^24^^,^^25^ In a study involving 887 patients undergoing major colorectal surgery, Ragg et al. reported that a high number of comorbidities was a risk factor for morbidity and mortality.^26^ Postoperative complications are known to be an important surgical factor affecting morbidity. In the present study, the rate of postoperative complications was also found to be significantly higher in Group 2 (P = 0.014) (Table 1). The rate of postoperative complications may have increased through reduction of the capacity to exert effort due to insufficient physical activity levels during the COVID period, diminished immunity due to advanced stage tumor and greater complexity of surgery.

In the literature, the rate of postoperative mortality has been reported to be 3-8%.^27^^,^^28^ The mortality rates in Group 1 and Group 2 of the present study were 5% and 8%, respectively, and these rates were consistent with findings in the literature. Although there was no significant difference in these mortality rates, mortality was high in Group 2 (Table 1).

In the present study, no significant increase in the pathological tumor stage (T) was seen during the pandemic. However, pathological lymph node (N) status was significantly higher (Table 2). In addition, liver metastasis was seen at a significantly higher rate in Group 2 (Table 2). According to these factors, the pathological stage of the patients in the pandemic was observed to be significantly more advanced (P = 0.005) (Table 2).

The Hartman procedure is frequently preferred in colorectal cancer surgery performed under emergency conditions.^29^ In parallel with the increase in the number of emergency surgeries during the pandemic period, we observed that colostomy was performed more often in Group 2 in our study. However, no significant difference was found between the groups (P = 0.390) (Table 2).

Individuals with suspicious findings relating to colorectal cancer disease may have preferred to wait at home and self-treat, instead of presenting to a healthcare institution. This may have been due either to limitations on the number of outpatient examinations and substantial selectivity regarding endoscopic and surgical procedures, or due to the precautions taken; or because of the fear of contracting COVID-19. This idea is supported by the observation that emergency operations on the symptoms manifested were performed more frequently and that tumors were only detected at more advanced stages.

## CONCLUSION

Through the COVID-19 pandemic, surgeons have been faced with patients presenting greater complications and disease at more advanced stages, due to delayed presentation at healthcare facilities.

In addition to irreversible complications due to these delays in presentation, the increased rates of morbidity and mortality, longer duration of hospital stay and higher patient costs also need to be taken into consideration.

More advanced social understanding is needed in order to address patients’ fears, in order to ensure that patients will promptly attend healthcare facilities in the event of a new COVID-19 wave with variant strains.

Moreover, if the course of colorectal cancers during such occurrences can be ascertained, policymakers and healthcare providers can become better organized with regard to disease management.
